# Meta-analysis and in-silico functional characterization of the *SNCA* variant rs356220 in Parkinson’s disease

**DOI:** 10.1038/s41598-025-04435-0

**Published:** 2025-07-02

**Authors:** Shradha Menon, Naushad Rais

**Affiliations:** School of Life Sciences, Manipal Academy of Higher Education, Dubai, United Arab Emirates

**Keywords:** rs356220, *SNCA*, Parkinson’s disease, PD, Meta-analysis, Computational analysis, Biochemistry, Computational biology and bioinformatics, Genetics, Neuroscience

## Abstract

The progression of Parkinson’s disease (PD) is influenced by genetic factors, particularly the Synuclein-Alpha (*SNCA*) gene, which encodes the alpha-synuclein (α-syn) protein involved in dopaminergic neuron degeneration. This study aimed to explore the relationship between rs356220 and PD risk and to understand its functional impact through computational analysis. We thoroughly reviewed nine databases regarding the association between this variant and PD risk. Firstly, a meta-analysis of 9 articles, consisting of 10 studies with 11,638 cases and 37,393 controls was conducted, that identified the C allele of rs356220 as a protective factor against PD (Odds Ratio (OR) 0.91, 95% Confidence Interval (CI): 0.88–0.94, P = 3.82E−08)). Subsequently, we characterized the functional impact of this non-coding variant in the pathophysiology of PD. In-silico process flow included transcription factor binding site (TFBS) analysis, pathway enrichment analysis, and protein interaction analysis. The TFBS analysis suggested that the C allele may influence multiple factors, while subsequent Pathway and Protein Network analyses identified proteins that enhance SNCA expression. Our investigation therefore reveals that rs356220 influences the dynamics of the α-syn protein through interactions with BAD, CANX, SLC18A1, and IRF1, potentially advancing the progression of PD. This research emphasizes the need for holistic study approaches to explore the intricacies of complex disorders like PD.

## Introduction

PD is a neurological disorder distinguished by a progressive deterioration of motor function, resulting in impairment and a reduction in the quality of life^1^. It is the second most prevalent neurodegenerative condition after Alzheimer’s disease (AD)^2–5^. PD is marked by the deterioration of dopaminergic neurons, primarily in the nigrostriatal pathway resulting in a range of motor and non-motor symptoms^6^. The clinical manifestations associated with this condition include resting tremors, rigidity, bradykinesia, and postural instability with non-motor symptoms such as mood disorders, cognitive impairment, sleep disorders, gastrointestinal symptoms, and pain^7–9^. The diagnosis of PD is typically considered to be a straightforward matter, often referred to as a "waiting room diagnosis." However, it is important to note that unambiguous signs and symptoms of the disease only become apparent at an advanced stage of its progression. It is anticipated that the global number of people diagnosed with PD will surpass 12 million by the year 2040^10^. The burden of PD will rise as a result of extended lifespans for both those with and without the condition^11^. According to the Michael J. Fox Foundation for Parkinson’s Research, symptoms of PD usually appear around the age of 60, and many people can live for 10 to 20 years following their diagnosis^12^. The precise cause of PD remains abstruse, although both genetic predisposition and environmental influences are thought to play a role in its development^13^.

**Fig. 1:**
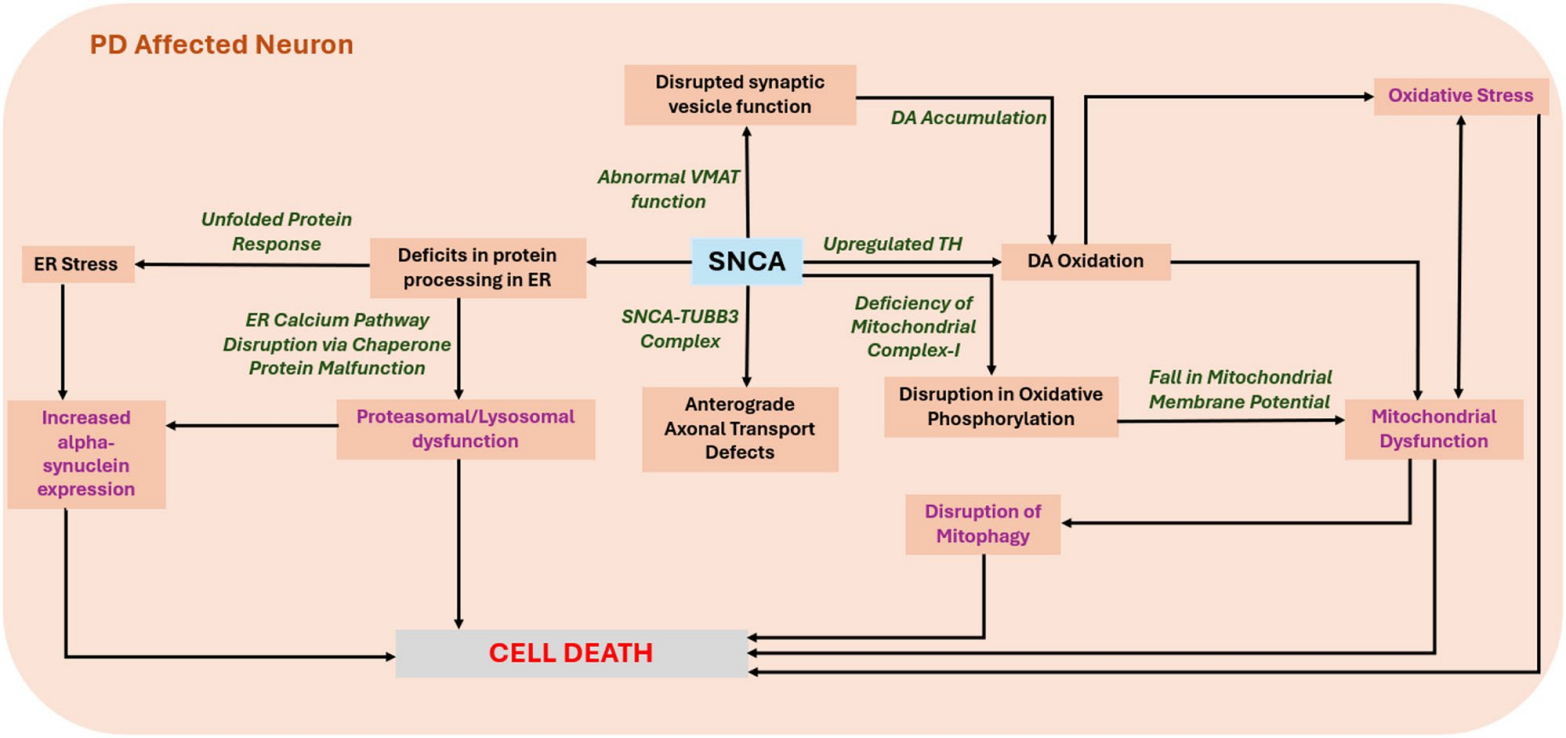
PD pathway displaying involvement of *SNCA* in substantia nigra extracted from KEGG Pathway Database. In PD, mutations in the SNCA gene play a role in mitochondrial dysfunction by inhibiting complex I, resulting in reduced ATP production and increased levels of reactive oxygen species (ROS). This process contributes to the acceleration of neurodegeneration. Also, they may also lead to elevated intracellular Dopamine (DA) levels, exacerbating DA toxicity and negatively affecting cognitive and motor functions. Additionally, these mutations have an impact on the 26S proteasome’s ability to degrade misfolded α-syn, leading to the activation of the Unfolded Protein Response (UPR), and an increase in ER stress. The degeneration of neuronal axons is another major consequence which can lead to the manifestation of abnormal motor symptoms.

Numerous studies have identified genetic loci associated with an increased risk of PD, such as SNCA, LRRK2, BST1, GBA, RIT2, HLA, and others^14^, of which *SNCA* is the gene of focus in this study. The *SNCA* gene is located on chromosome 4q22 and consists of six components spanning approximately 114 kb. The final 5 segments of the gene responsible for its translation encode a small protein α-syn with a molecular weight of 14.5 kDa and comprising 140 amino acids^15–18^. The protein is thought to have a role in the formation of SNARE protein complexes, which are responsible for vesicle fusion, and in the regulation of neurotransmitters^19^. α-syn is typically concentrated in synapses, where it is believed to play a role in the functioning of synaptic vesicles. In addition to its crucial role in synapse function, α-syn also possesses numerous biological functions as illustrated on pathways generated by KEGG Pathway Database (Fig. 1)^20–23^.

These include the induction of apoptosis, elevation of oxidative stress, regulation of Ca^2+^ and mitochondrial homeostasis, and the occurrence of cell cycle aberrations Burré, Sharma and Südhof, 2018. Mitochondrial dysfunction has been recognized as detrimental in PD, with research indicating a link between the impairment of mitochondrial complex I and the neuronal degeneration caused by ATP deficiency in the striatal substantia nigra^24^. Furthermore, the autophagy pathway of the ubiquitin–proteasome system plays a role in the dysfunction of both lysosomes and proteasomes^25,26^.

Understanding the function of disease-related genetic variations is a major challenge in translational genomics currently, as the majority of these variations found through Genome Wide Association Studies (GWAS) are located in non-coding areas of the genome^27^. One such variant is *SNCA* variant rs356220, located ~ 5 kb downstream of the gene in the 3ʹ untranslated region (3ʹUTR)^28^, which has been identified as a genetic variant that elevates the susceptibility to PD in global populations^29,30^. Interestingly, in a Chinese population, this SNP has been reported to have a protective effect as well^31,32^. In a study conducted by Guo et.al in 2014, the researchers specifically focused on the SNPs rs2736990 and rs356220 in *SNCA*, which were found to be protective factors against Early-Onset PD. However, these variants did not show any association with other clinical features of PD, including sex, onset symptoms (tremor or rigidity), cognitive impairment, anxiety, and depression^31^. Shahmohammadibeni et al. conducted a study in the Iranian population, which added more support to the idea that rs356220 is a risk variant associated with PD^33^.

Notably, rs356220 signifies a broader category of non-coding functional variants with strong associations to disease, yet the underlying biological mechanisms remain inadequately understood^34^, representing a significant challenge in post-GWAS interpretation^35^. Despite the complexities involved, it is crucial to uncover the origins of disease susceptibility and how these disease-associated variants elevate risk^36^. The predictive interplay of SNPs and their functional effects may be related to the biological processes that trigger, develop, progress, or exacerbate the disease. Hence, transitioning from mere statistical correlation to functional analysis is crucial in understanding disease pathogenesis^27^.

In the present study, we have updated a large meta-analysis of previously reported studies to confirm the association of rs356220 with the PD risk across global populations. In addition, we also performed computational analysis to investigate the underlying effects of this non-coding variant on disease progression.

## Materials and methods

### Meta-analysis

#### Literature search and data extraction

In order to conduct a comprehensive meta-analysis, a thorough literature search was performed, encompassing the PubMed NIH (https://pubmed.ncbi.nlm.nih.gov/), Scopus (https://www.scopus.com/home.uri) and the Google Scholar Interface (https://scholar.google.com/) databases. The search was carried out using specific keywords such as ‘SNCA’, ‘SNCA variants’, ‘PD’, ‘Parkinson’s Disease’, 'Alpha-Synuclein’, ‘rs356220’. Filters for ‘All’ and 'Meta-Analysis’ were applied to ensure the relevance and reliability of the retrieved studies. The search covered the period from 2005 to 2023 to include recent publications.

To gather general quantitative data on the genetic variant, SNPnexus database (https://www.snp-nexus.org/v4/) was used. To validate the data obtained, information from other repositories such as dbSNP NIH database (https://www.ncbi.nlm.nih.gov/snp/), SNPedia (https://www.snpedia.com/index.php/), NHGRI-EBI GWAS Catalog (https://www.ebi.ac.uk/gwas/variants/rs356220), PharmGKB pharmacogenomic database (https://www.pharmgkb.org/) and Varsome Clinical software (https://varsome.com/variant/hg19/rs356220) were consulted, providing valuable insights into the polymorphism under investigation.

It was determined that information regarding the variant is presently lacking in PharmGKB and is classified as not clinically significant by dbSNP. On the contrary, the SNP is noted to be associated with an increased risk for PD, exhibiting an odds ratio of 1.5 according to the SNPedia repository. This association is further supported by findings from GWAS studies conducted by the NHGRI-EMBL GWAS and Varsome Clinical Databases.

We assessed a total of 9 candidate gene case–control articles that aligned with the research objective in order to gather the required data (Table 1). The authors compiled information on the first author’s name, publication year, the specific population being studied, the ethnicity of the cases and control groups, as well as the total number of cases and controls involved in the study, including their data on this genetic variant and Hardy–Weinberg Equilibrium (HWE) test results. For more information, please refer to Supplementary Table S1.

**Table 1:** Case–control publications included in the meta-analysis of SNCA variant rs356220.

Sl. no	Reference	Population	Country	PD/control
1	Mueller et al.^37^	Caucasian	Germany	669/1002
2	Pankratz et al.^38^	Caucasian	America	445/335
3	Hamza et al.^39^	Caucasian	America	1458/931
4	Do et al.^40^	Mixed	Multi-country	3400/29,000
5	Trotta et al.^41^	Caucasian	Italy	904/891
6	Miyake et al.^42^	East Asian	Japan	229/357
7	Guo et al.^31^	East Asian	China	1011/721
8	Hill-Burns et al.^43^	Caucasian	Multi-country	2000/1986
9	Shahmohammadibeni et al.^33^	West Asian	Iran	520/520

#### Inclusion and exclusion criteria

**Fig. 2:**
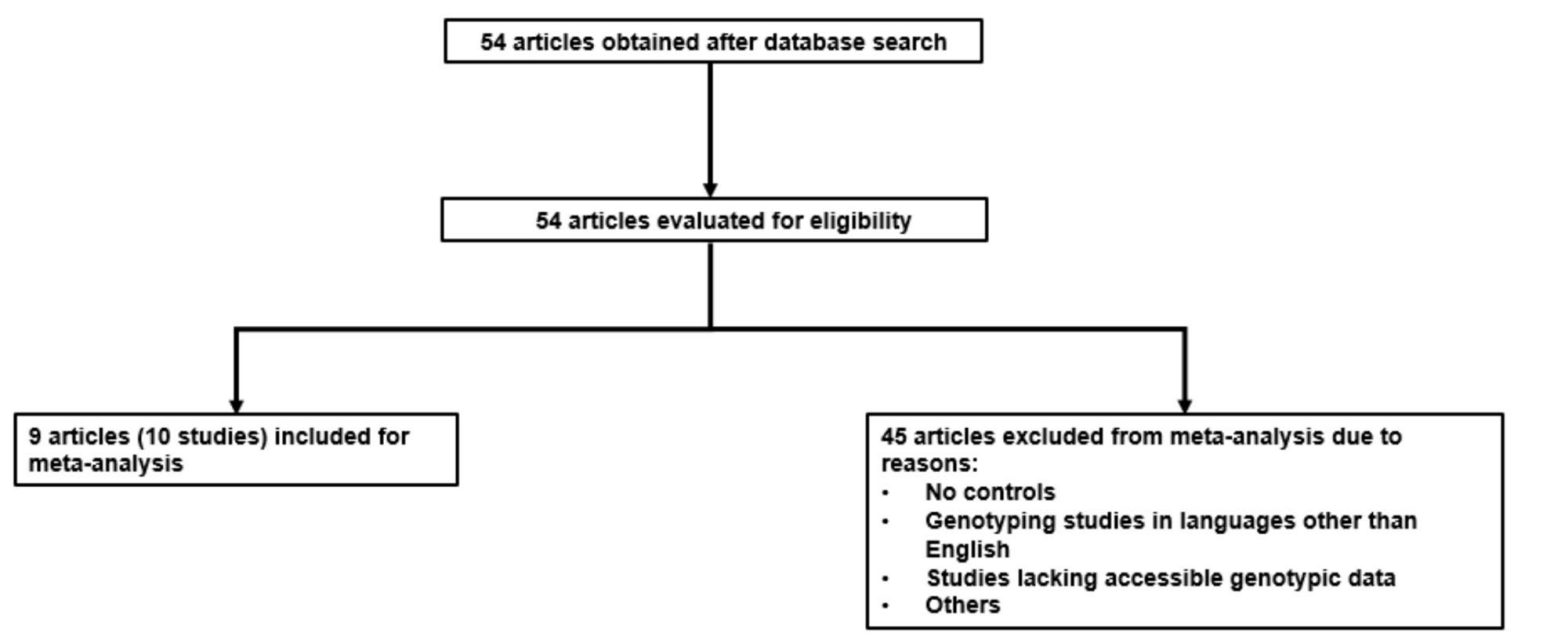
Search flowchart for selected polymorphisms and PD. Out of 54 articles that were identified and evaluated, 9 met the inclusion criteria and were chosen for meta-analysis, while 45 articles were excluded based on the specified exclusion criteria outlined in the flowchart.

In order to be included, the studies must adhere to specific criteria as indicated in Fig. 2.

The publications that meet these criteria should consist of studies that primarily investigate the correlation between PD and other factors, such as case–control studies involving healthy controls, as well as cohort genotyping studies. Assorted studies were excluded, due to several reasons, such as being studies that solely focused on PD cases without including any control groups, systematic reviews that did not provide accessible genotypic data, genotyping studies conducted in languages other than English, and studies that involved Parkinsonian syndromes and other synucleopathies.

#### MetaGenyo

Meta-analysis was carried out utilizing MetaGenyo, a user-friendly web application. This tool facilitated the computation of various essential parameters in the meta-analysis process, including association values such as ORs with 95% CI for allelic, dominant and recessive models as well as HWE test, assessment of heterogeneity, and evaluation of publication bias indicators^44^. For all statistical tests, a p-value equal to 0.05 was considered as level of significance. Forest plots were created only for the total population and no subgrouping was done due to a smaller sample size and limited diversity in ethnicity. Tests for heterogeneity were estimated by the Q test and its derivative I^2^ test and were shown in the context of C as the risk allele for rs356220. Heterogeneity p-values were computed and if the value fell below 0.10, it signified the presence of heterogeneity^45^. I^2^ values of 40% or less may suggest insignificant heterogeneity, while values between 30 and 60% may indicate moderate heterogeneity. Values between 50 and 90% may suggest substantial heterogeneity, and values between 75 and 100% may imply considerable heterogeneity^46^. The Tau^2^ Estimate serves as a measure of the overall level of heterogeneity present. H^2^ represents a test statistic that quantifies the proportion of overall variability attributed to heterogeneity. It illustrates the relative discrepancy between the observed Q and its anticipated value when heterogeneity is not present. Funnel plots and Egger’s test for each genetic model were analyzed to evaluate publication bias^47^. If the p-value from Egger’s regression test falls below 0.05, there is a potential presence of publication bias in the meta-analysis^48^.

### In-silico functional analysis of rs356220

**Fig. 3:**
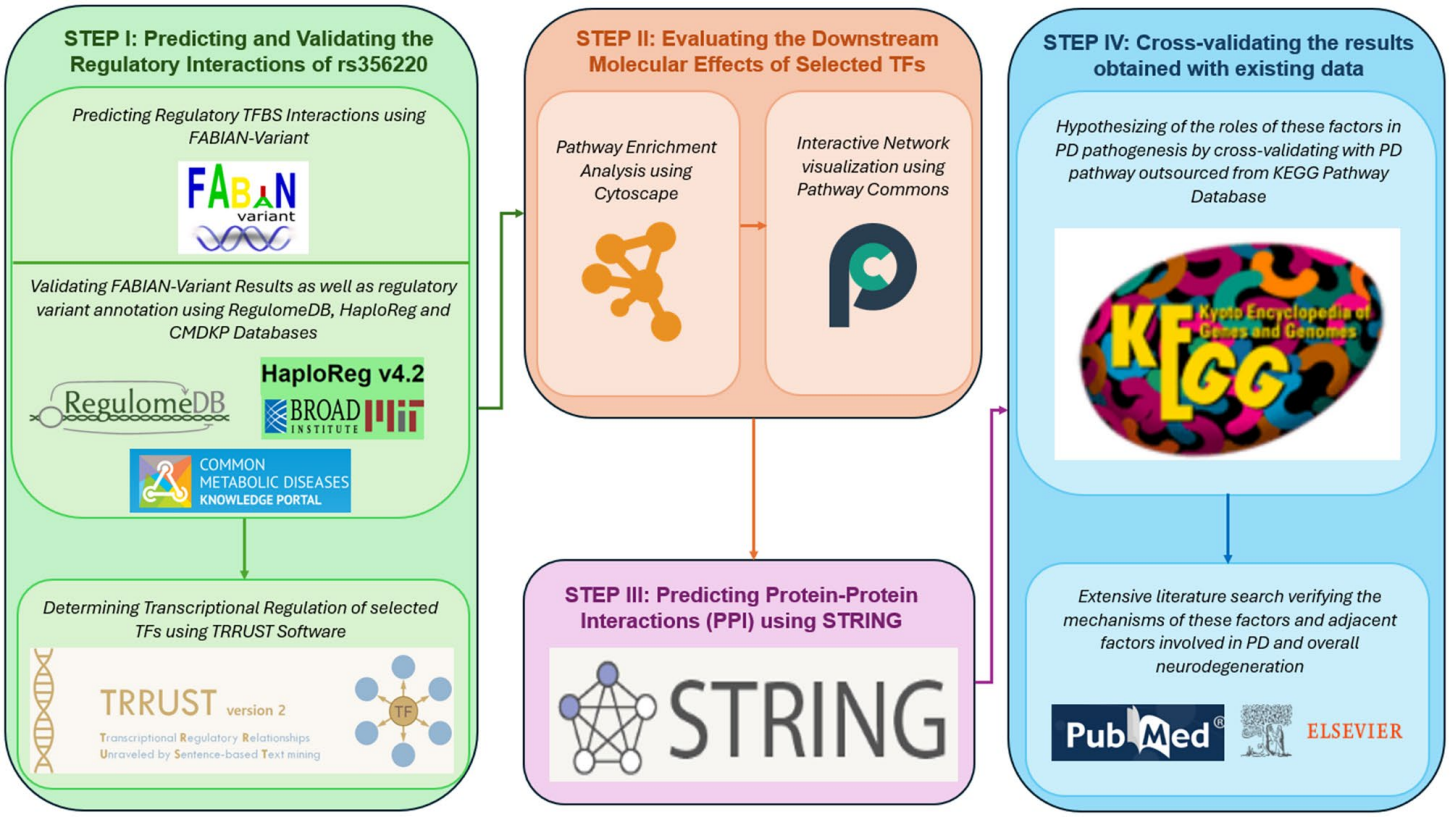
In-silico functional analysis process flow diagram.

We conducted a computational functional analysis that examined the downstream effects of the rs356220 SNP on SNCA expression and its involvement in PD pathogenesis, the workflow of which has been illustrated (Fig. 3).

#### Predicting and validating the regulatory interactions of rs356220

To perform an in-silico functional analysis, we used a recently developed tool, FABIAN variant^49^ to determine the impact of rs356220 (hg38- 4:89720189T>C) on various TFBS (Fig. 3). This web-based application uses transcription factor flexible models (TFFMs) and position weight matrices (PWMs) to predict the degree to which SNPs are likely to create or disrupt the TFBS. The tool uses only the TFFM for analysis as it provides a better representation of the predictions when compared with PWMs. The FABIAN application provides one score per TFBS per variant which varies from 1 to − 1, with 0 indicating no disruption in binding. A higher score demonstrates an increased binding affinity, and a lower score indicates a weakened binding affinity with an arbitrary cut-off score of ± 0.8 to select those that we deemed to be ‘high confidence’ predictions^36,49^.

TFBS results cross-validated using 3 additional databases such as RegulomeDB v2.2^50^, HaploReg v4.2^51^, Common Metabolic Diseases Knowledge Portal (CMDKP)^52^ (Fig. 3).

RegulomeDB provides vital insights into non-coding single nucleotide variants in the human genome, including rsid, peaks, prediction rank, score, and allelic frequency data from GnomAD, 1000 Genomes, and TOPMED^50^. The browser displays a scoring system indicating evidence strength for each variant (1–7), with lower scores suggesting higher functionality likelihood. Additionally, RegulomeDB’s probability score ranges from 0 to 1, with 1 indicating a high likelihood of being a regulatory variant. This composite tool also integrates DNase-seq, transcription factor ChIP-seq, and chromatin state segmentations obtained from ENCODE and Roadmap Epigenomics, covering a diverse range of cell types^50,53^, which we have used to analyze this regulatory variant.

HaploReg, developed by the BROAD Institute of MIT, is a valuable web tool for analyzing non-coding genome annotations in GWAS^51^. Our analysis concentrated on transcription factor (TF) motif examination. The results leverage cross-referenced PWM models derived from both reference and alternative sequences to estimate TF binding affinities. Positive scores reflect an increased probability of TF binding, whereas negative scores indicate a diminished likelihood. This tool carried out a comprehensive annotation of the SNP by leveraging Roadmap Epigenomics and ENCODE chromatin state maps, histone modification profiles, and DNase hypersensitivity data to identify enhancer marks that overlap with rs356220.

The CMDKP database validates the impact of rs356220 on TFBS. The results page displays predictions from the Ensembl Variant Effect Predictor categorizing variant consequences and predicted TF binding motifs from HaploReg at the variant position in hg37 genome format (4:90641340:T:C). Additional details such as the PWM and the influence of the alleles on the TF binding motif are also included. The PWM column provides PWM for the affected motif. The Delta value indicates the difference between Reference and Alternate scores, reflecting the alternate allele’s impact. The Position and Strand specify the variant’s location within the TF binding motif and the DNA strand (plus or minus), respectively. The Reference and Alternate scores measure the influence of the respective alleles on the TF binding motif.

To ascertain the regulatory interactions of selected transcription factors affected by rs356220, we utilized the Transcriptional Regulatory Relationships Unraveled by Sentence-based Text mining (TRRUST) v2.0 database^54^, informed by the TFBS results (Fig. 3). TRRUST v2.0 is a resource that offers insights from current literature and generates new hypotheses on transcriptional regulation in human diseases^54^. It includes curated interactions of human TFs and their regulatory mechanisms, whether activation or repression. This tool stands apart from the others in its focus. It is specifically designed to validate TF interactions and identify new interactions following the identification of TFs, rather than assessing the influence of SNPs on TF binding sites. This approach allowed us to clarify the relationships and the implications of these TFs.

#### Evaluating the downstream molecular effects of selected transcription factors

Online tools, Cytoscape^55^ and Pathway Commons^56,57^ were used to construct the gene network and analyze the regulatory impact factors (Fig. 3). Using Cytoscape 3.10.3 (https://cytoscape.org/) to corroborate pathway interactions of SNCA and its associated transcription factors affected by the non-coding variant rs356220 supplements TFBS analysis by providing enhanced visualization, integrating diverse biological data, enabling network analysis, and cross-referencing with known pathway interactions.

Pathway Commons is a tool that consolidates and shares data on metabolic and signaling pathways, genetic interactions, and gene regulation networks. When searching for genes, the platform uses this data to create an interactive network visualization called the Interactions app.

The search query for both Cytoscape and Pathway Commons involved 7 factors—SNCA, BAD, CANX, SLC18A1 and IRF1 (Fig. 3). These putative and hypothetical interactions were cross-referenced with their involvement with PD pathogenesis mechanisms illustrated in the KEGG database (Fig. 1)^20–23^.

#### Predicting protein–protein interactions (PPI)

Finally, PPI were predicted by STRING (Fig. 3) which serves as a comprehensive repository of both anticipated and established PPIs. These interactions encompass both direct physical connections and indirect functional associations. They are derived from a variety of sources, including computational predictions, knowledge transfer across different organisms, and the aggregation of interactions from primary databases^58^. The query search involved 5 proteins—SNCA, BAD, CANX, SLC18A1, and IRF1. PPI analysis using the STRING Database investigates both hypothetical and established interactions involving proteins encoded by factors affected by rs356220 . This adds significant value by revealing how proteins interact within broader regulatory networks, thereby enhancing the insights gained from TFBS and pathway enrichment analyses.

## Results

### Meta-analysis

In total, nine publications were assessed for rs356220. The isolated genotypic data for rs356220 provided information on a total population of 11,638 cases and 37,393 controls. The allelic model (OR 0.91, 95% CI (0.88–0.94)), dominant model (OR 0.89, 95% CI (0.85–0.95)), and recessive model (OR 0.88, 95% CI (0.83–0.93)) in the Fixed Effects model demonstrate a statistically significant relationship between the genetic variant and PD (Table 2), that C allele has a moderately protective correlation.

**Table 2:** Meta-analysis summary table of correlation between variant rs356220 and PD.

Model	Number of studies	Inverse variance-fixed effects model			Inverse variance -random effects model		
		OR	95% CI	P	OR	95% CI	p
Allele contrast (C vs. T)	10	0.91	[0.88; 0.94]	3.82E-08	0.98	[0.76; 1.27]	0.883
Recessive model (CC vs. CT + TT)	10	0.88	[0.83; 0.93]	2.330E-06	1.15	[0.86; 1.55]	0.345
Dominant model (CC + CT vs. TT)	10	0.89	[0.85; 0.95]	< 0.00001	0.92	[0.64; 1.31]	0.628
pairw1 (CC vs. TT)	10	0.86	[0.80; 0.92]	3.27812E−05	1.10	[0.72; 1.70]	0.651
pairw2 (CC vs. CT)	10	0.91	[0.86; 0.97]	0.002	1.25	[0.98; 1.60]	0.070
pairw3 (CT vs. TT)	10	0.91	[0.86; 0.97]	0.002	0.86	[0.62; 1.20]	0.378

Conversely, the results from the Random Effects model do not show a statistically significant association (Table 2). For more information, please refer to Supplementary Figs. S1–3.

This discrepancy underscores the uncertainty surrounding the fundamental link between this variant and disease.

Heterogeneity tests, especially as per I^2^, exhibit moderate to high heterogeneity (I^2^ equal to or greater than 50%) for overall populations and the results of Egger’s test indicated (Table 3) that there was no significant bias in this meta-analysis. Conversely, funnel plots revealed asymmetry and hence publication bias caused by unknown factors (Figs. S4–6).

**Table 3:** Heterogeneity test results and publication bias for rs356220.

Model	Heterogeneity					publication bias
	Tau^2^	H	I^2^	Q	p-value	p-value (Egger’s test)
rs356220						
Allele contrast (T vs. C)	0.16	6.74	0.98	408.90	0	0.46
Recessive model (TT vs. TC + CC)	0.21	4.63	0.95	192.82	0	0.05
Dominant model (TT + TC vs. CC)	0.32	5.86	0.97	308.79	0	0.86

### In-silico functional analysis

FABIAN-Variant online tool identified 328 transcription factors possibly influenced due to *SNCA* variant, rs356220, out of which we selected the factors, BAD (BCL2 Associated Agonist of Cell Death), CANX (Calnexin), SLC18A1 (Solute Carrier Family 18 Member A1) indicating extreme disruption in binding as per the cut off values mentioned previously.

Therefore, FABIAN-Variant assigned loss of binding scores of transcription factors, SLC18A1 (Combined score of -0.5260), CANX (Combined score of -0.3829), while BAD (Combined score of + 0.9139) was assigned a gain of binding score (Table 4).

**Table 4:** TFBS results from FABIAN variant.

Highly impacted positive binding factors [(+ 0.9139 to + 0.1512)]	Low impacted positive binding factors [(+ 0.1489 to + 0.0501)]	Highly impacted negative binding factors [(− 0.7742 to − 0.1554)]	Low impacted negative binding factors [(− 0.1427 to − 0.0503)]
1. BAD*	1. PKNX1	1. RUNX1	1. ZN652
2. DUS3L	2. ESR1	2. RUNX3	2. HOXD10
3. PPP5C	3. HES6	3. NKX3-2	3. ZNF211
4. EXO5	4. ZN320	4. IRF2	4. ZN691
5. PRKRIR	5. TYY1	5. SLC18A1*	5. ONECUT3
6. IRF9	6. ZNF320	6. ZNF354C	6. HMBOX1
7. RBM17	7. OTX1	7. RPL35	7. NR3C2
8. ZNF384	8. NEUROD2	8. RUNX2	8. NR6A1
9. MSI2	9. ZNF713	9. CANX*	9. POU2F1
10. RFC3	10. ZNF324	10. MXD4	10. HNRNPLL

*Selected factors for further investigation in this study.

In order to validate the TFBS data from FABIAN-Variant, we examined transcription factor binding motifs information sourced from the RegulomeDB, Haploreg, and CMDKP databases. A single transcription factor, IRF1 (Interferon Regulatory Factor 1), was identified as being consistently impacted across all these repositories, with a combined PWM TFBS score of + 0.2603 in FABIAN-Variant.

To unravel transcriptional regulatory relationships between the selected TFs and *SNCA*, we used TRRUST database. Interestingly, the TRRUST database did not contain any information on SNCA, CANX or SLC18A1. Additionally, it implied that BAD is not a TF. Only IRF1 was clearly found to be a TF.

So, we proceeded to perform a pathway enrichment analysis using robust in-silico tools such as Cytoscape and Pathway Commons to establish a functional correlation between these factors and SNCA expression. The results of Pathway Commons predicted that of the factors, BAD binds with SNCA. Cytoscape provided visual connection in the form of signaling pathway which clearly supplemented that SNCA binds BAD. Enrichment analysis revealed individual divergent effects and crosstalk of mechanisms due to effects of BAD and SNCA with scores less than 0.05 (Tables 5 and 6) that could potentially lead to neurodegeneration when cross-referenced with the KEGG database PD pathway interactome (Fig. 1).

**Table 5:** BAD pathway enrichment analysis table.

Description	Evidence codes	Intersecting genes	p-value	Precision	Recall	Source	Term ID	Term name
"A heterodimeric protein complex consisting of BAD and BCL-2, members of the Bcl-2 family of anti- and proapoptotic regulators." [GOC:so, PMID:14634621]	“IPI”	BAD	0.0199	1	0.5	Gene Ontology Cellular Component branch	GO:0097138	BAD-BCL-2 complex
"Any process that activates or increases the frequency, rate or extent of glucokinase activity, the catalysis of the transfer of a phosphate group, usually from ATP to a glucose molecule." [GOC:mah]	“ISS”	BAD	0.0495	1	1	Gene Ontology Biological Process	GO:0033133	Positive regulation of glucokinase activity
"Any process that activates or increases the frequency, rate or extent of intrinsic apoptotic signaling pathway in response to osmotic stress." [GOC:BHF, GOC:mtg_apoptosis, GOC:rl, GOC:TermGenie, PMID:14569084]	“IEA”	BAD	0.0495	1	1	Gene Ontology Biological Process	GO:1902220	Positive regulation of intrinsic apoptotic signaling pathway in response to osmotic stress

**Table 6:** SNCA pathway enrichment analysis table.

Description	Evidence codes	Intersecting genes	p-value	Precision	Recall	Source	Term ID	Term name
mmu-miR-153-3p	“MIRNA”	SNCA	0.0088	1	0.33	mirTarBase miRNA targets	MIRNA:mmu-miR-153-3p	mmu-miR-153-3p
"Any process that increases the frequency, rate or extent of assembly of the SNARE complex." [GOC:rb]	“IDA”	SNCA	0.0495	1	1	Gene Ontology Biological Process	GO:0035543	Positive regulation of SNARE complex assembly
Any process that stops, prevents, or reduces the frequency, rate or extent of the directed movement of dopamine into a presynaptic neuron or glial cell. [GOC:ai]	“IDA”	SNCA	0.0495	1	1	Gene Ontology Biological Process	GO:0051585	Negative regulation of dopamine uptake involved in synaptic transmission
"Any process that stops, prevents, or reduces the frequency, rate or extent of the directed movement of norepinephrine into a cell." [GOC:ai]	“IDA”	SNCA	0.0495	1	1	Gene Ontology Biological Process	GO:0051622	negative regulation of norepinephrine uptake
"Any process that stops, prevents, or reduces the frequency, rate or extent of the directed movement of catecholamine neurotransmitters into a neuron or glial cell." [GOC:ai, GOC:dph, GOC:tb]	“IDA”	SNCA	0.0495	1	1	Gene Ontology Biological Process	GO:0051945	Negative regulation of catecholamine uptake involved in synaptic transmission
"Any process that modulates the frequency, rate or extent of glutathione peroxidase activity." [GO_REF:0000059, GOC:bf, GOC:PARL, GOC:TermGenie]	“IDA”	SNCA	0.0495	1	1	Gene Ontology Biological Process	GO:1903282	Regulation of glutathione peroxidase activity
"Any process that activates or increases the frequency, rate or extent of glutathione peroxidase activity." [GO_REF:0000059, GOC:bf, GOC:PARL, GOC:TermGenie, PMID:23507046]	“IDA”	SNCA	0.0495	1	1	Gene Ontology Biological Process	GO:1903284	Positive regulation of glutathione peroxidase activity
DJ-1-SNCA complex, high molecular weight complex	“CORUM”	SNCA	0.0499	1	0.5	Manually annotated protein complexes from mammalian organisms	CORUM:5830	DJ-1-SNCA complex, high molecular weight complex
SNCA-TUBB3 complex	“CORUM”	SNCA	0.0499	1	0.5	Manually annotated protein complexes from mammalian organisms	CORUM:7241	SNCA-TUBB3 complex

**Fig. 4:**
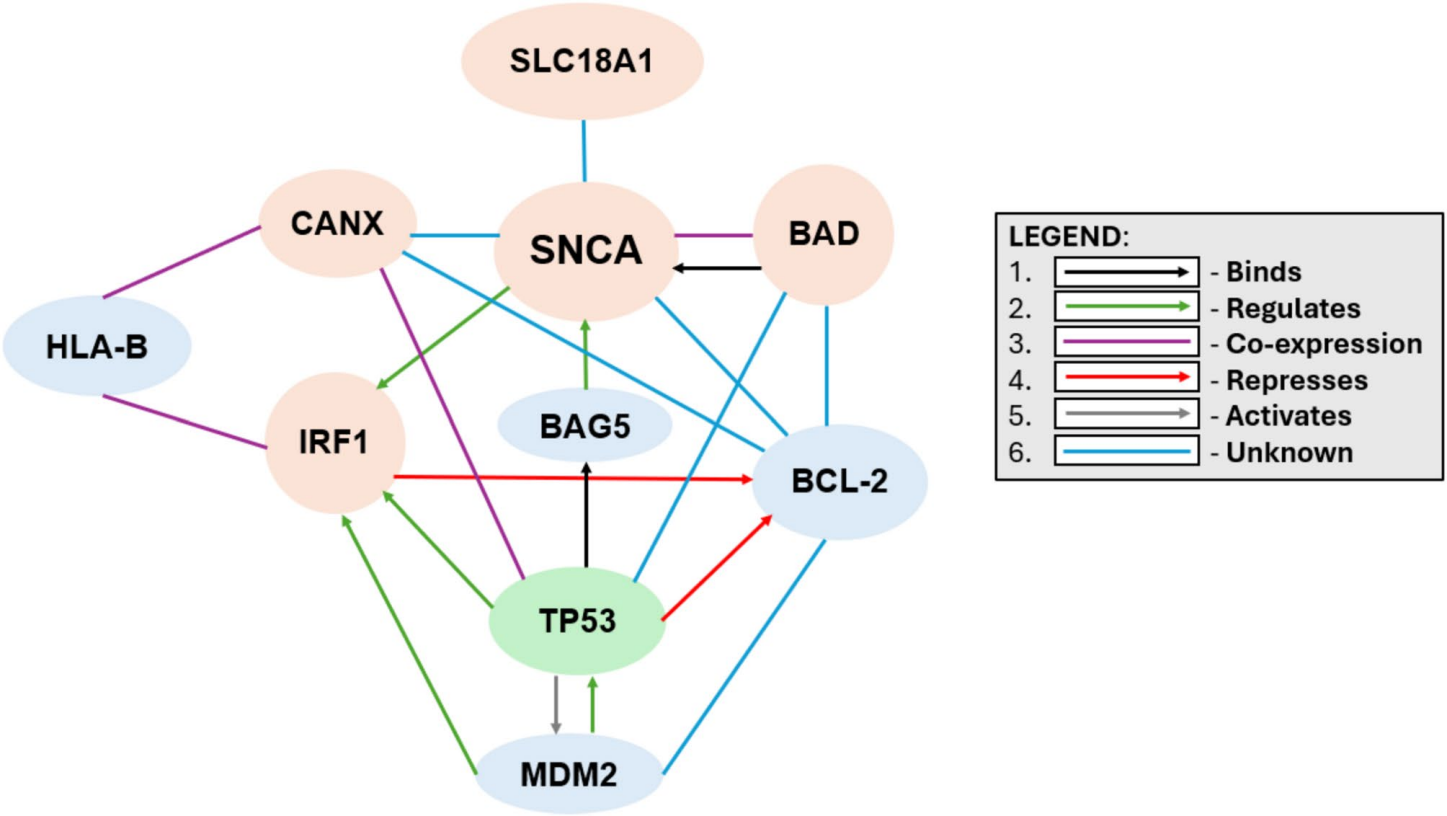
PPI diagram. Peach colored proteins are the ones selected and discussed in the study in association with SNCA. TP53 is also emphasized here since it is at the intermediary of almost all the factors in this network. Blue colored proteins are the extended associated factors.

Notably, search results of all other implied factors did not reveal any implied neuronal interaction with SNCA expression in Cytoscape and Pathway Commons. The PPI analysis supports the pathway enrichment findings on the binding and co-expression of BAD and SNCA. SLC18A1 interaction analysis shows a strong interaction with SNCA, hinting at potential consequences of SNCA dysfunction. Additionally, CANX analysis reveals direct links to SNCA and involvement in a network involving BCL-2 and TP53, potentially impacting CANX functionality. Our results also suggest that TP53 may contribute to neuronal loss, forming a triad with BAD and BCL-2. STRING analysis shows that p53 (activate TP53) inhibits BCL-2, while BAD and BCL-2 interact in various ways. p53 might function as part of a three-component system alongside IRF1 and Mdm2, indicating that p53 and Mdm2 play a role in regulating IRF1. Additionally, there seems to be a reciprocal regulatory interaction between p53 and Mdm2, forming a feedback loop. Lastly, the analysis indicates that p53 modulates the expression of SNCA, with the BAG5 chaperone protein facilitating this regulation (Fig. 4).

Additionally, regulatory annotations of rs356220 using RegulomeDB v2.0 revealed a rank of 5 and a functional score of 0.00143, indicating moderate regulatory potential. It overlaps six DNase peaks and is situated within heterochromatin regions of various non-neural tissues, including mesenchymal stem cells and fibroblasts. Conversely, in brain and neurosphere-derived cells, it aligns with a quiescent chromatin state, demonstrating evidence of chromatin accessibility and weak enhancer signals in areas such as the midbrain and substantia nigra.

HaploReg analysis further supports the regulatory potential of rs356220, revealing overlaps with enhancer-associated histone marks (H3K4me1, H3K27ac) in various brain tissues and neuronal progenitor cells. This variant also influences transcription factor binding motifs and is associated with multiple PD GWAS signals, underscoring its relevance in a neurological context.

## Discussion

The identification of genetic variability at *SNCA* can be considered as the most dependable correlation between a common genetic risk locus and PD discovered thus far^59^. From a causal standpoint, the amplification of the *SNCA* gene locus results in elevated quantities of the normal α-syn protein, thereby giving rise to familial PD^60,61^. The hindered removal of α-syn can lead to increased levels of the protein in sporadic cases of PD. Additional comprehensive analyses have revealed a correlation between genetic diversity in other regions of *SNCA* and susceptibility to the disease, specifically in the latter half of the gene’s 3ʹ end^37,62,63^. It is important to highlight that transcripts with longer 3′ UTRs are more commonly found in brain tissue, indicating that variations in this specific region may exert a more noteworthy influence on the central nervous system^64^.

Each successive GWAS revealed that PD is linked to a growing array of SNPs characterized by diminishing effect sizes^65,66^. The significance of the outcomes derived from these associations has been the subject of extensive discussion^67,68^. The impetus for this discussion stems from the ambiguity regarding the mechanisms that underlie these associations. Notably, over 90% of the risk SNPs are located outside of protein-coding regions, primarily within putative regulatory elements that influence the expression of genes that are frequently not well characterized^69^.

Previous research has documented the link between *SNCA* rs356220 and the risk of PD. Nevertheless, the findings presented in various publications remain inconclusive. Although a significant correlation between SNCA rs356220 and PD has been identified in both Caucasian and Asian groups^33,37,41,42^, it has also been observed to reduce the likelihood of PD in the Chinese population^31^. Our research involved a meta-analysis of existing studies to explore the relationship between rs356220 and PD risk worldwide. The original comprehensive quantitative analysis was published in the year 2020^70^ and the study is facing a 5-year literature gap. We have incorporated additional association studies to supplement the existing extensive data.

We enhanced the quality of our analysis by including new and reliable though not conclusive, data on the correlation between 3ʹ-UTR *SNCA* polymorphism rs356220 and the risk of PD across global populations of Asian and Caucasian ethnicities. The meta-analysis conducted on 9 articles aimed to investigate the relationship between the C allele of rs356220 and PD, with ambiguity displayed in statistical correlation analysis results (Table 2). The protective effect of the C allele was observed in the Fixed Effects model, which assumes that all studies included in the meta-analysis estimate the same true effect size. However, when the Random Effects model was applied, which accounts for potential heterogeneity among the studies, yielded no statistically significant association (Table 2). This suggests that the effect of the variant may vary across different populations or study designs. Factors contributing to this discrepancy may encompass variations in ancestry, genotyping methodologies, and disparities in study sizes.

Significant strengths exist in the current study. The 2020 meta-analysis^70^ encountered various limitations that we successfully addressed. Initially, the insufficient number of studies and sample size hindered the ability to provide substantial evidence on the correlation between *SNCA* rs356220 polymorphism and PD risk. The meta-analysis analyzed data from six articles, encompassing 5333 PD cases and 5477 controls. However, our analysis expanded by including three additional articles, totaling 11,638 cases and 37,393 controls, significantly enhancing the statistical power of our study. The funnel plot analysis in the previous meta-analysis was limited due to the inclusion of only six articles. In our updated meta-analysis, we included 9 articles improving the reliability of the funnel plot analysis. Additionally, we utilized the Egger’s test instead of Begg’s test, which provided more accurate results and helped us detect publication bias more effectively. Our study introduced a new perspective by considering the mutant C allele as the allele associated with PD, contrary to previous literature. Nonetheless, our study does have certain limitations, specifically the small sample size that lacks sufficient power to detect the effects. Incorporating more homogeneous sub-cohorts could enhance the effectiveness of PD genetic association studies, particularly concerning obscure non-coding variants like rs356220^71^. There were some excluded studies from this meta-analysis as well due to inaccessibility of genotyping data (Fig. 2).

Our hypothesis focused on the potential role of the *SNCA* non-coding downstream variant rs356220 in affecting α-syn protein function and advancing PD development. Studies have suggested a link between rs356220 and rigidity, indicating that rs356220 loci may play a part in the emergence of motor symptoms characteristic of PD^72^. Nevertheless, there is a lack of pertinent pharmacogenomic information regarding this SNP in existing databases. Consequently, this raises the question of how the impact of a non-coding regulatory SNP, such as rs356220, translates to the molecular pathogenesis of PD. Furthermore, it prompts the question of why rs356220 is frequently linked to the global prevalence of PD, despite being classified as a benign variant with no established pharmacogenomic significance. Therefore, we conducted an extensive analysis utilizing in-silico tools.

We assessed the changes in transcription factor binding brought on with the presence of SNP rs356220 utilizing a newly developed in-silico application, FABIAN-Variant^49^, which demonstrated gain of binding of factors BAD and IRF1 and loss of binding of factors, CANX and SLC18A1 (Table 4) with respect to binding affinity to *SNCA* gene. Pathway enrichment analysis indicated a binding interaction between BAD and SNCA. Subsequent protein-level analysis uncovered a network of interrelated factors, including BAD, CANX, and IRF1, associated with SNCA. TP53 is identified as a central protein that mediates the interactions among these factors. SLC18A1, however, seems to be in direct involvement with SNCA.

Cross-validating all these interactions with the KEGG PD schematic (Fig. 1), it can now be implied that SLC18A1 dysfunction directly leads to SNCA dysfunction at the transcription level and could potentially promote DA toxicity and impaired neurotransmission, leaving dopaminergic neurons vulnerable to damage at the protein level. FABIAN-Variant results revealed the loss of binding of SLC18A1, also known as Vesicular Monoamine Transporter 1 (VMAT1) because of the presence of rs356220 (Table 4) and STRING database functional network analysis uncovered a potential PPI. Although, it was reported initially that only Vesicular Monoamine Transporter 2 (VMAT2) is expressed in brain, recent studies indicate that VMAT1 is also expressed in brain, thus making both transporters plausible candidate genes for neuropsychiatric disorders, such as PD^73,74^. However, further investigation using Cytoscape and Pathway Commons did not yield a direct confirmatory correlation between VMAT1 and SNCA.

**Fig. 5:**
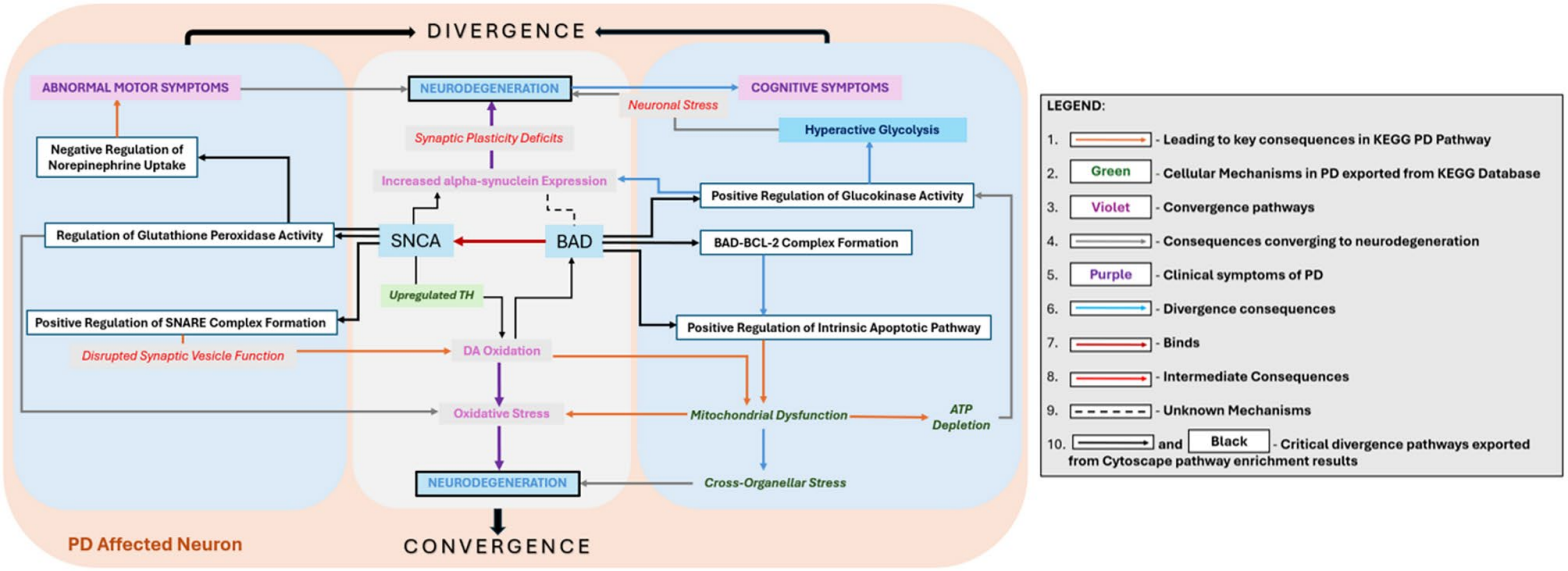
The Convergence-Divergence Model shows the combined effects of SNCA and BAD. Using Cytoscape Software, we identified their interactions, leading to neuronal death through oxidative stress and apoptosis. BAD exacerbates neuronal stress by potentially enhancing glycolytic and promoting cell death through the destabilization of mitochondrial integrity and an increase in oxidative stress in PD. Additionally, upregulated DA synthesis produces ROS that impair mitochondrial function and synaptic dynamics. These divergent disruptions could translate to motor and cognitive deficits. Both SNCA and BAD converge upon oxidative stress, leading to neurodegeneration.

Secondly, gain of binding of BAD could potentially affect multiple SNCA regulation networks at the transcription level. Research indicates that elevated levels of α-syn can suppress protein kinase C (PKC) function^75^. PKC enzymes are key in neurotransmitter release, synaptic plasticity, and stress responses. Their dysregulation is linked to neurodegenerative diseases like AD^76^. Therefore, the interaction of α-syn with BAD and the inhibition of PKC activity suggest that increased α-syn expression may have harmful effects on neurons. However, according to the TRRUST database^54^, BAD is not a TF but is rather regulated by TFs. The nature of the interaction is still not fully understood, as pathway analysis performed in Cytoscape indicates that BAD interacts with SNCA in the α-syn pathway. Furthermore, the pathway enrichment results for BAD reveal possible upregulation of intrinsic apoptosis (Table 5), resulting in mitochondrial dysfunction due to fall of mitochondrial membrane potential and ROS toxicity (Fig. 1). This leads to increased dependency of neurons on glycolysis as BAD indirectly results in upregulation of glucokinase (GK) activity (Table 5), possibly trying to compensate for the consequent ATP insufficiency and high cellular stress in neurons^77^. This suggests that GK could serve as a potential biomarker for neurodegenerative conditions. BAD also exhibits proapoptotic effects in neurons^78^ by interacting with anti-apoptotic BCL-2 (Table 5), as well as responds to cellular stress signals from TP53 (Fig. 4). Therefore, BAD could induce neurodegeneration through aberrant activation of the intrinsic apoptotic pathway or as a product of mitochondrial dysfunction due to DA oxidation and this has been evident in recent studies^79,80^. Spatial learning and memory could also be impacted through its direct mediation of neuronal apoptosis and indirect promotion of neuroinflammation^81^. The results of the SNCA enrichment analysis show that SNCA has several important effects (Table 6). First, it positively regulates the assembly of the SNARE complex, which impacts neurotransmitter release and synaptic function. Second, it influences the uptake of DA and norepinephrine, which could potentially lead to motor dysfunction. Lastly, it forms a crucial complex with DJ-1, a gene closely associated with PD, triggering oxidative stress and contributing to the loss of dopaminergic neurons (Table 6). Pathway interactome analysis indicates that SNCA significantly impacts neurodegeneration via mitochondrial impairment and DA imbalance, often associated with BAD co-expression. The interaction data obtained from Pathway Commons further reinforces the results from Cytoscape, indicating that BAD does bind with SNCA. As a result, we propose this potential interaction, which may contribute to neurodegeneration in PD through various mechanisms, illustrated in a proposed convergence-divergence model (Fig. 5).

CANX loss of binding could influence various SNCA regulatory interactions at the transcription level (Table 4), leading to a cascade of consequences. Studies suggest that α-syn may interact with ER chaperones, including CANX, disrupting proteostasis mechanisms and delaying cargo export from the ER^82,83^. This disruption may lead to the buildup of misfolded α-syn, which can cause stress in ER and Golgi apparatus. According to a study, the malfunction of CANX initiates ER stress, activating the UPR and associated cellular phenomena, including apoptosis^84^. In PD models, mitochondrial oxidant stress leads to oxidized DA accumulation, disrupting lysosomal function, and promoting α-syn buildup^85^. Studies have also suggested an indirect interaction between *SNCA*, *LRRK2*, and *CANX* in PD, with implications for ER stress and cell death in astrocytes^86^. CANX was found to interact with BCL-2, a regulator of Ca^2+^ transport across the ER membrane (Fig. 4), though its precise role in modulating intracellular Ca^2+^ levels is still debated^87^. Additionally, CANX also appears to affect immune response due to reduced antigen presentation as well as immunoproteasome integrity^88^. PPI analysis additionally implied CANX to be co-expressed with HLA-B (MHC-1) protein (Fig. 4). CANX is an integral component of the MHC class I antigen processing and presentation machinery (APM)^89^. Therefore, it can be posited that the impairment of APM components like CANX may compromise the integrity of the immunoproteasome, leading to reduced antigen presentation, promoting acceleration of disease progression^88^. IRF1 is also associated with immune dysregulation just like CANX, albeit more directly. At the transcription level, SNCA dysregulation and TFBS analysis of rs356220 has been understood with IRF1 being a primary factor implied as a direct genetic consequence in PD. At the PPI level, IRF1 was found to interact with HLA-B subsequently affecting immune activation and response (Fig. 4). Research conducted by Sulzer et.al has indicated that PD could potentially be classified as an autoimmune disorder^90^. A recent study found that α-Syn increases IRF-1 expression, triggering a neuroinflammatory response by inhibiting MDM2-mediated ubiquitylation, leading to higher IRF-1 protein levels (Fig. 4)^91^. However, our TFBS analysis has implied some level of transcriptional alteration between SNCA and IRF1. IRF1 was also found to be directly involved in SNCA regulation by influencing microglial activation and clearance. A significant discovery from a 2021 study indicated that the buildup of α-Syn in microglial cells resulted in a marked increase in the expression of genes related to (i) essential components of the inflammasome pathway such as Casp1, (ii) inflammatory cytokines like IL-18, and (iii) crucial factors in inflammatory nuclear transcriptional pathways, such as IRF1^92^. This is a clear indicator of neuroinflammation in PD^93^.

TP53 is a key factor in these protein interactions (Fig. 4), with p53 activation contributing to neurodegeneration through mechanisms such as mitochondrial dysfunction, excessive mitochondrial Ca^2+^ accumulation, ROS generation, abnormal protein aggregation, and impaired mitophagy^94^. In PD patients and animal models, increased p53 expression in the substantia nigra is linked to dopaminergic neuron degeneration^95,96^. PPI analysis from STRING suggests that activated p53 is part of a theoretical three-component system with BAD and BCL-2, though the interaction between p53 and BAD is unclear. They also indicate that p53 may inhibit BCL-2, with various factors influencing the PPI between BCL-2 and BAD (Fig. 4). Additionally, p53 interacts with IRF1 and MDM2^97^, mutually regulating each other, and affects SNCA expression through the BAG5 chaperone protein, highlighting its role in the protein interactome and significance in PD (Fig. 4). Stress activates BAG5 chaperone expression through p53 interaction with its promoter. However, excessive stress may cause overproduction of BAG5, potentially contributing to α-syn aggregation (Fig. 4). Induced BAG5 has been shown to interact with α-syn in cell cultures and brain lysates from PD patients^98^. Furthermore, cytosolic p53 reduces the ubiquitin-dependent degradation of the α-syn protein^99^. According to STRING Database results, CANX also shares a connection with TP53 (Fig. 4). A study conducted in 2016^100^ revealed that the activated oncoprotein p53 facilitates the progression of metastatic tumors through downstream effectors, including ENTPD5, an ER UDPase that plays a role in the calnexin/calreticulin cycle, a critical process involved in the proper folding and maturation of N-glycoproteins, which are essential for various cellular functions. This interaction was validated by a 2023 study^101^. These findings support the proposed link between CANX and p53. Consequently, therapeutic strategies that rely on p53 may offer promising targets for the development of new neuroprotective medications for PD^102^.

**Fig. 6:**
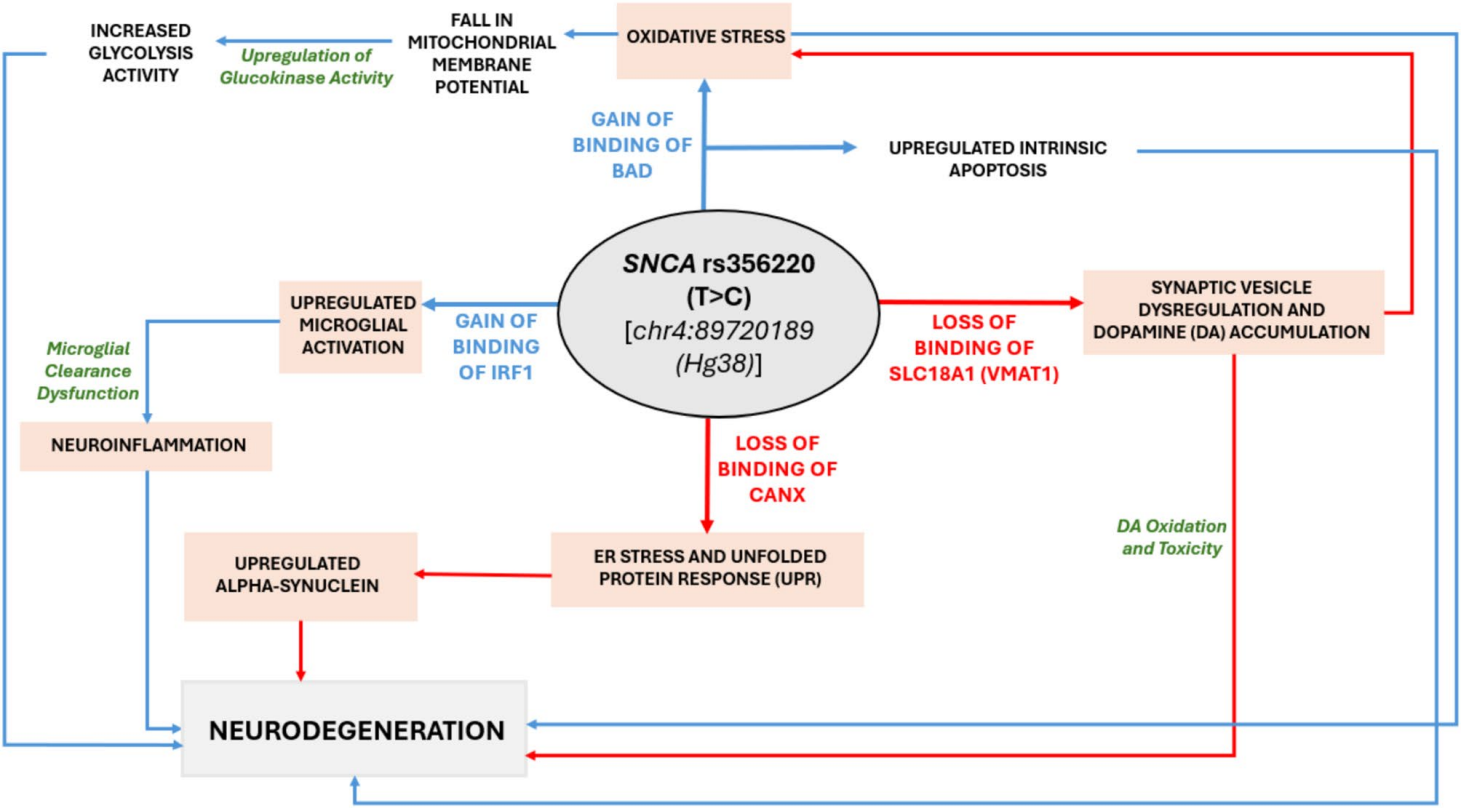
The proposed schematic summarizes the extensive molecular ramifications of the non-coding 3ʹUTR SNP rs356220, which plays a role in the pathogenesis of PD. It indicates that the upregulation of α-syn expression is not the primary outcome of this SNP; instead, it represents an additional effect that contributes to neurodegeneration. This illustration emphasizes pathways in which heightened α-syn levels worsen mitochondrial dysfunction and oxidative stress, both of which are critical elements in PD pathology. These stress responses significantly contribute to neurodegeneration and arise not merely from protein aggregation but from a more extensive response induced by increased α-syn. Furthermore, the metabolism of DA itself produces ROS as byproducts. The interplay between oxidative stress resulting from DA metabolism and mitochondrial dysfunction fosters a detrimental environment that particularly impacts dopaminergic neurons.

Our findings therefore provide valuable information on potential biomarkers and mechanisms associated with the neuropsychiatric symptoms and cognitive deterioration observed in this condition (Fig. 6). This research thus reinforces the emerging agreement that aggregation represents a downstream occurrence or an epiphenomenon, rather than the fundamental cause of neuronal death in PD.

Consequently, our computational results raise the possibility that the C allele of rs356220 could affect molecular pathways associated with PD possibly via nuanced regulatory or compensatory mechanisms. These may include cell-specific processes^103^. This is becoming increasingly of significance, as the biological functions of cells such as neurons^104^ with their synaptic dynamics^105^ from those of glial cells^106^, which involve roles in neuronal maintenance, toxicity clearance and blood–brain barrier interactions^107^. Both cell types have been affected in PD pathogenesis^105,108^. Furthermore, secondary factors may be triggered by pathomechanisms like ER stress or neuroinflammation, which could impact neurodegeneration and the phenotypic trajectory of the disease^109,110^.

Although this interpretation seems to differ from previous findings that associated the T allele with a higher risk of PD^31,33,37,40,42,43,111,112^, it emphasizes the complex, allele-specific regulatory impacts that can result from non-coding variants. This research does not negate the potential harmful effects of the T allele; instead, it underscores the functional importance of rs356220 as a regulatory SNP that may have implications for disease, depending on cellular context and molecular processes.

Epigenetic regulation may also modulate the effects of this variant. According to chromatin state and regulatory annotations from RegulomeDB, rs356220 has limited regulatory activity in most tissues, primarily found in heterochromatin or quiescent states in mesenchymal stem cells, fibroblasts, heart, liver, and vasculature. Nevertheless, its overlap with DNase hypersensitive sites and weak H3K4me1 and H3K27ac enhancer marks in neural progenitor cells and brain regions, such as the midbrain and substantia nigra, suggests a notable regulatory function related PD, especially regarding SNCA expression and regulation.

Haploreg findings further indicate that rs356220 is located within active or poised enhancer regions in the brain, supporting the notion of potential cell-type-specific regulation near SNCA. The convergence of enhancer histone modifications, altered transcription factor motifs, and associations identified in PD-related GWAS validates the variant’s significance in neuronal regulatory networks. In addition, changes in chromatin dynamics could shift enhancer interactions from SNCA to other genes linked to PD, potentially exacerbating the condition.

Collectively, these findings bolster the argument that non-coding variants influence disease risk through tissue-specific enhancer activity during disease^113,114^. This also reinforces the case for regulatory SNPs requiring functional validation in neural-specific models. Additionally, this study suggests that rs356220 may exert more than a neutral regulatory role, potentially contributing to deleterious downstream effects. It also displays an incongruency between genotype and phenotype with respect to this non-coding variant, which may help explain the inconsistencies reported across previous genetic association studies in PD.

Nonetheless, our research has notable limitations. The reliance on bulk post-mortem brain tissue hinders the identification of specific cell types, potentially obscuring unique regulatory effects in vulnerable populations like dopaminergic neurons^115^. Furthermore, the effects specific to cell types add complexity to the analysis^103^. Post-mortem samples can introduce confounding variables like RNA degradation and differing post-mortem intervals, which can affect transcript integrity and expression profiles^116^. Bulk-tissue eQTL analyses tend to average expression changes across all cell types, potentially concealing opposing regulatory influences^117^. Utilizing single-cell expression datasets from various brain regions could uncover these contrasting patterns, elucidating why a variant might repress in one scenario yet favor neurodegeneration in another. Moreover, examining DNA methylation and histone modifications surrounding the SNP in post-mortem PD and control brains through bisulfite sequencing and ChIP-seq would enhance our understanding of the epigenetic landscapes across different genotypes^118^. In summary, the integration of functional assays and multi-omics strategies in forthcoming studies will be essential in processing the broader implications of these non-coding variants in PD pathology.

## Conclusion

We conducted this study to investigate the role of a non-coding variant in the development and progression of PD as previous studies only emphasized the risk association of this variant with the disease. This is bolstered by a comprehensive meta-analysis and bioinformatic assessment of underlying biological mechanisms. In-silico functional analyses have identified rs356220 as a potentially deleterious SNP associated with PD. We propose that the pathogenesis of PD is characterized by a complex interplay of various molecular interactions, involving critical factors such as BAD, CANX, IRF1, and SLC18A1, suggesting that the mechanisms underlying neuronal loss are more intricate than previously recognized. The downstream effects of rs356220 on TFBS modifications related to SNCA, along with the potential PPI pathways implicated in PD development, highlight the significance of regulatory dysfunctions that may play a role in neurodegeneration. Identifying the rs356220 SNP in patients may help stratify genetic risk for PD, enabling early diagnosis and preventive measures.

## Supplementary Information

Supplementary Information 1.

Supplementary Information 2.

## Data Availability

Data is provided within the manuscript or supplementary information files.
