# Supplementary material for: Meta-analysis and in-silico functional characterization of the SNCA variant rs356220 in Parkinson’s disease
Source: Sci Rep. 2025 Jul 2;15:23358. doi: 10.1038/s41598-025-04435-0 (PMC12223105; doi:10.1038/s41598-025-04435-0)
Supplement: Supplementary file 2 — Supplementary Information 2. [file 41598_2025_4435_MOESM2_ESM.docx]

**Meta-Analysis and In-Silico Functional Characterization of the *SNCA* Variant rs356220 in Parkinson’s Disease**

**Shradha Menon and Naushad Rais**

**School of Life Sciences, Manipal Academy of Higher Education, Dubai, United Arab Emirates**

**SUPPLEMENTARY DATA**

**Table S1: Genotypic data extracted from case-control studies used for meta-analysis of rs356220.**

| **Author** | **Population** | **CC**  **cases** | **CT**  **cases** | **TT**  **cases** | **CC**  **controls** | **CT**  **controls** | **TT**  **controls** | **HW-**  **P-value** | **HW-adjusted P- value** |
| --- | --- | --- | --- | --- | --- | --- | --- | --- | --- |
| Mueller et. al, 2005 (A) | Germany | 66 | 167 | 107 | 83 | 310 | 287 | 0.9597 | 0.9597 |
| Mueller et.al, 2005 (B) | Germany | 70 | 163 | 966 | 46 | 152 | 124 | 0.9578 | 0.9597 |
| Pankratz et. al, 2009 | America | 91 | 212 | 142 | 42 | 172 | 121 | 0.1099 | 0.3663 |
| Hamza et. al, 2010 | America | 383 | 984 | 633 | 256 | 916 | 814 | 0.946 | 0.9597 |
| Do et. al, 2011 | Multi-Country | 1094 | 1713 | 618 | 11792 | 13789 | 4014 | 0.8653 | 0.9597 |
| Miyake et.al, 2012 | Japan | 100 | 97 | 32 | 126 | 174 | 57 | 0.8116 | 0.9597 |
| Trotta et.al, 2012 | Italy | 156 | 422 | 326 | 123 | 409 | 359 | 0.7055 | 0.9597 |
| Burns et.al, 2014 | Multi-Country | 493 | 770 | 302 | 813 | 901 | 272 | 0.3747 | 0.9368 |
| Guo et.al, 2014 | China | 343 | 482 | 186 | 193 | 383 | 145 | 0.0714 | 0.357 |
| Shahmohammadibeni et. al, 2016 | Iran | 93 | 246 | 181 | 52 | 254 | 214 | 0.0617 | 0.357 |

**Forest plots of the association between rs356220 and PD risk under allele, dominant and recessive models among overall populations (11638 cases and 37393 controls)**

**Inverse Variance-Fixed Model**

**
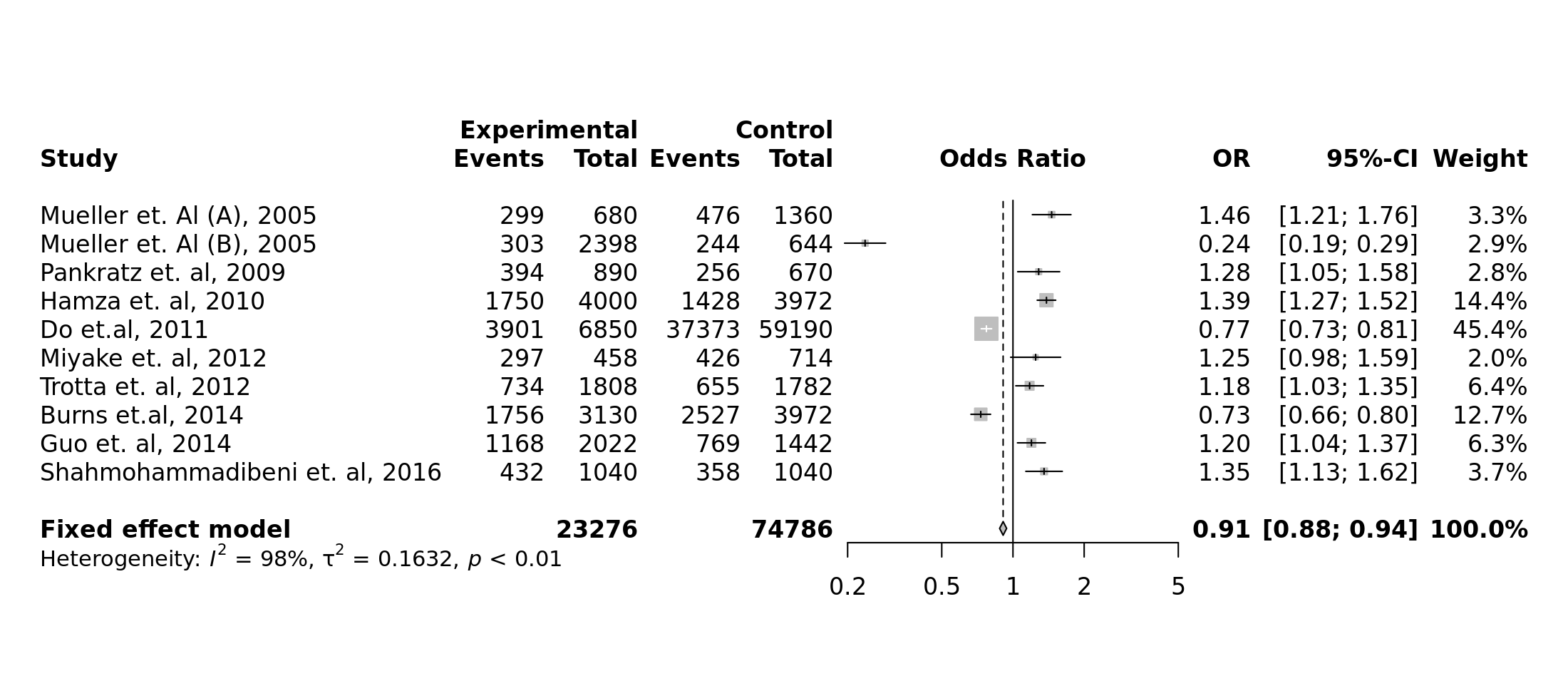
Figure S1. Allele Model**

**
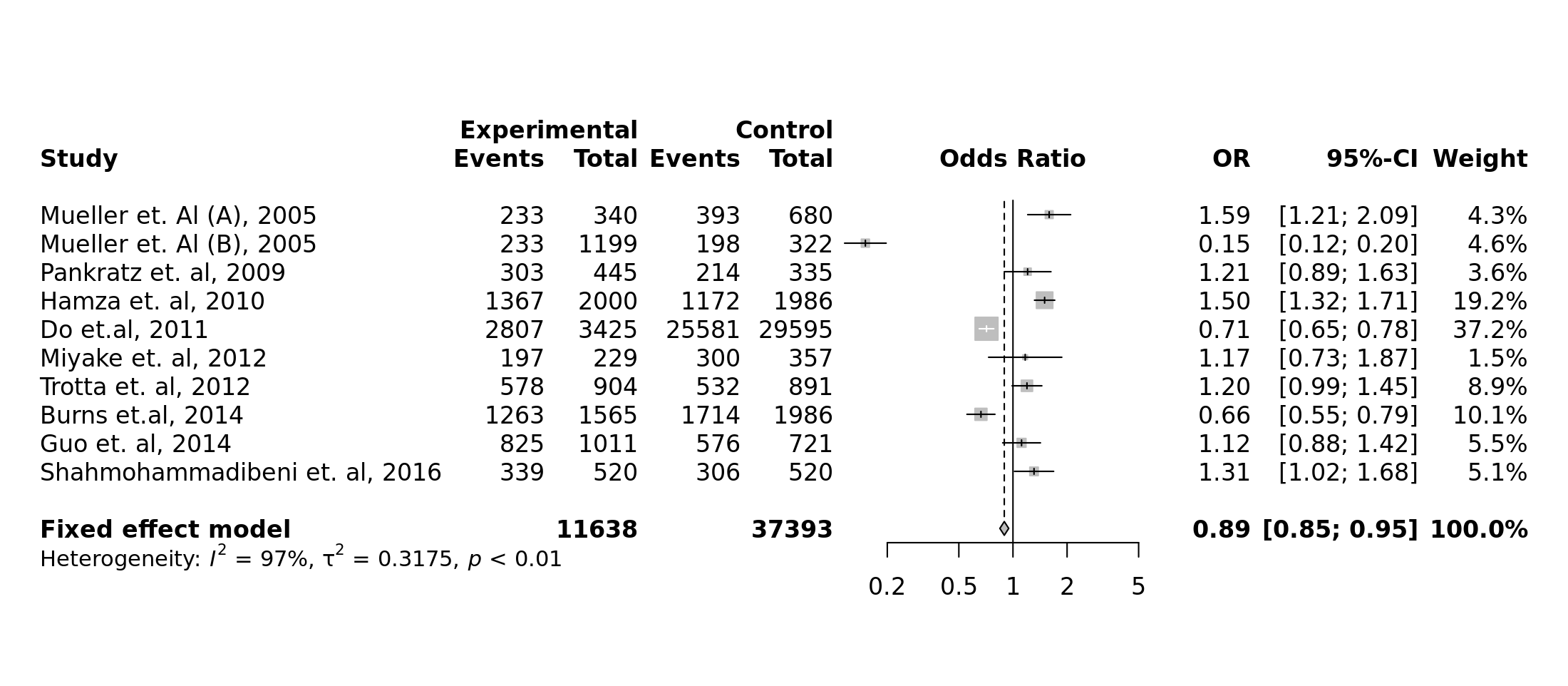
Figure S2. Dominant Model**

**
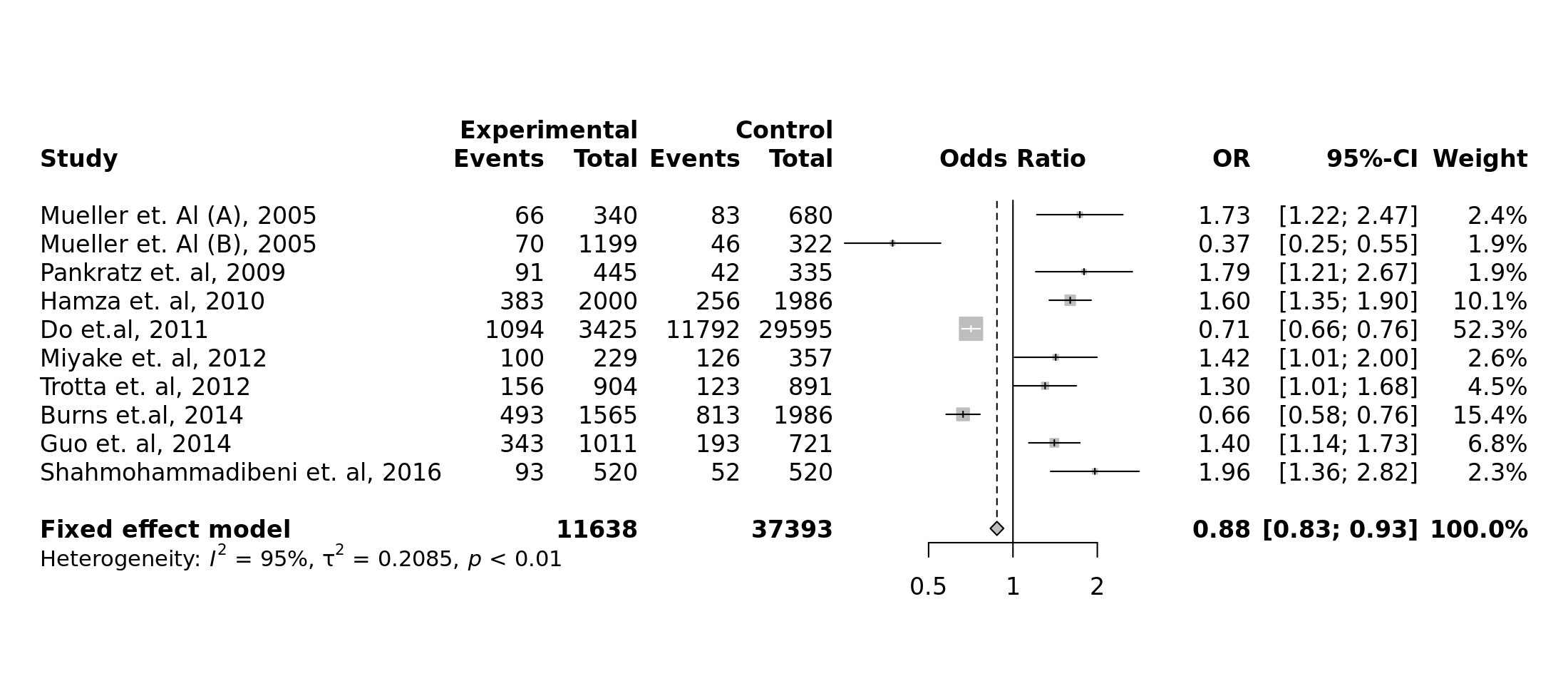
**

**Figure S3. Recessive Model**

**
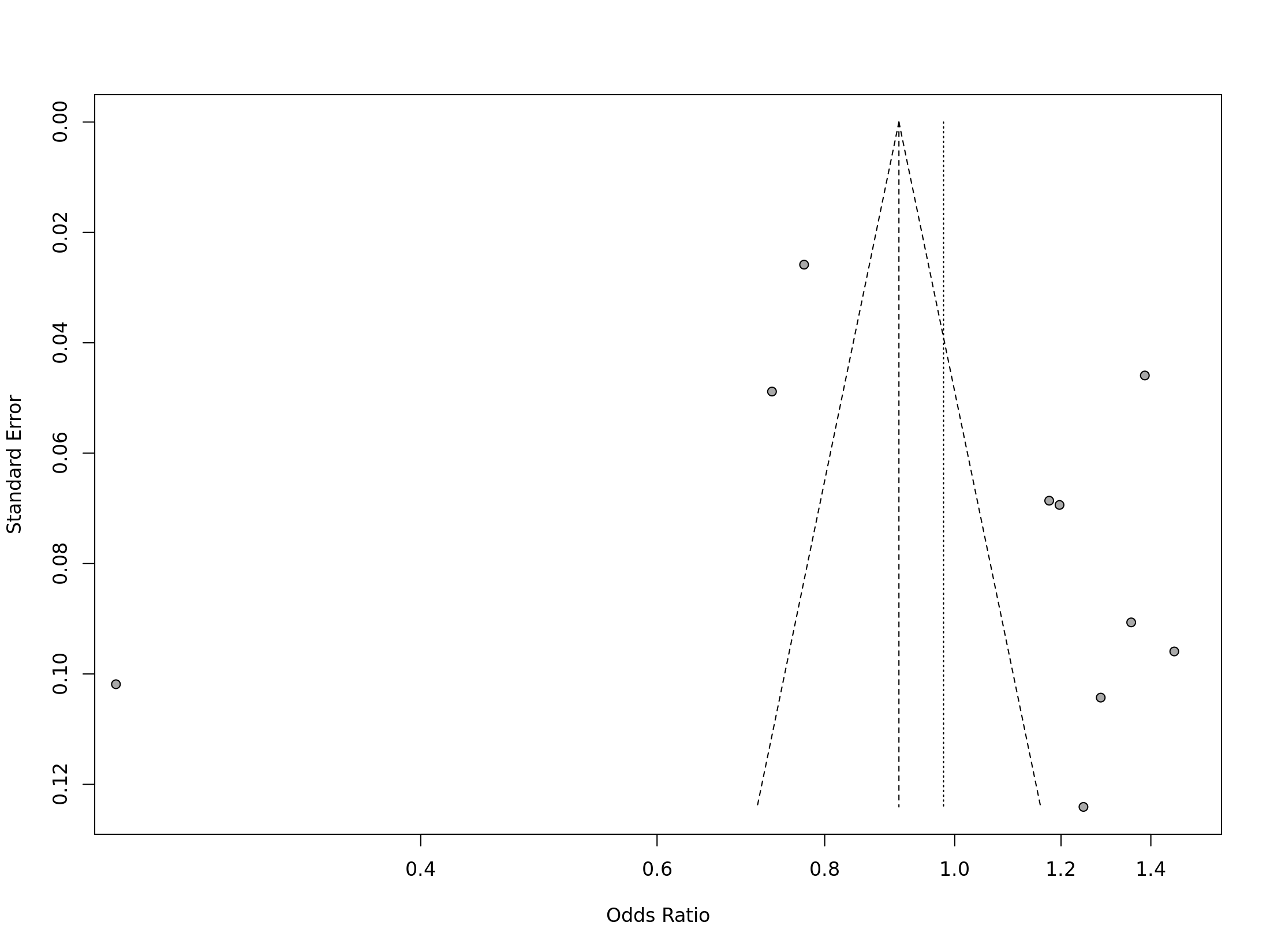
**

**Figure S4. Funnel plot of rs356220 allelic model**

**
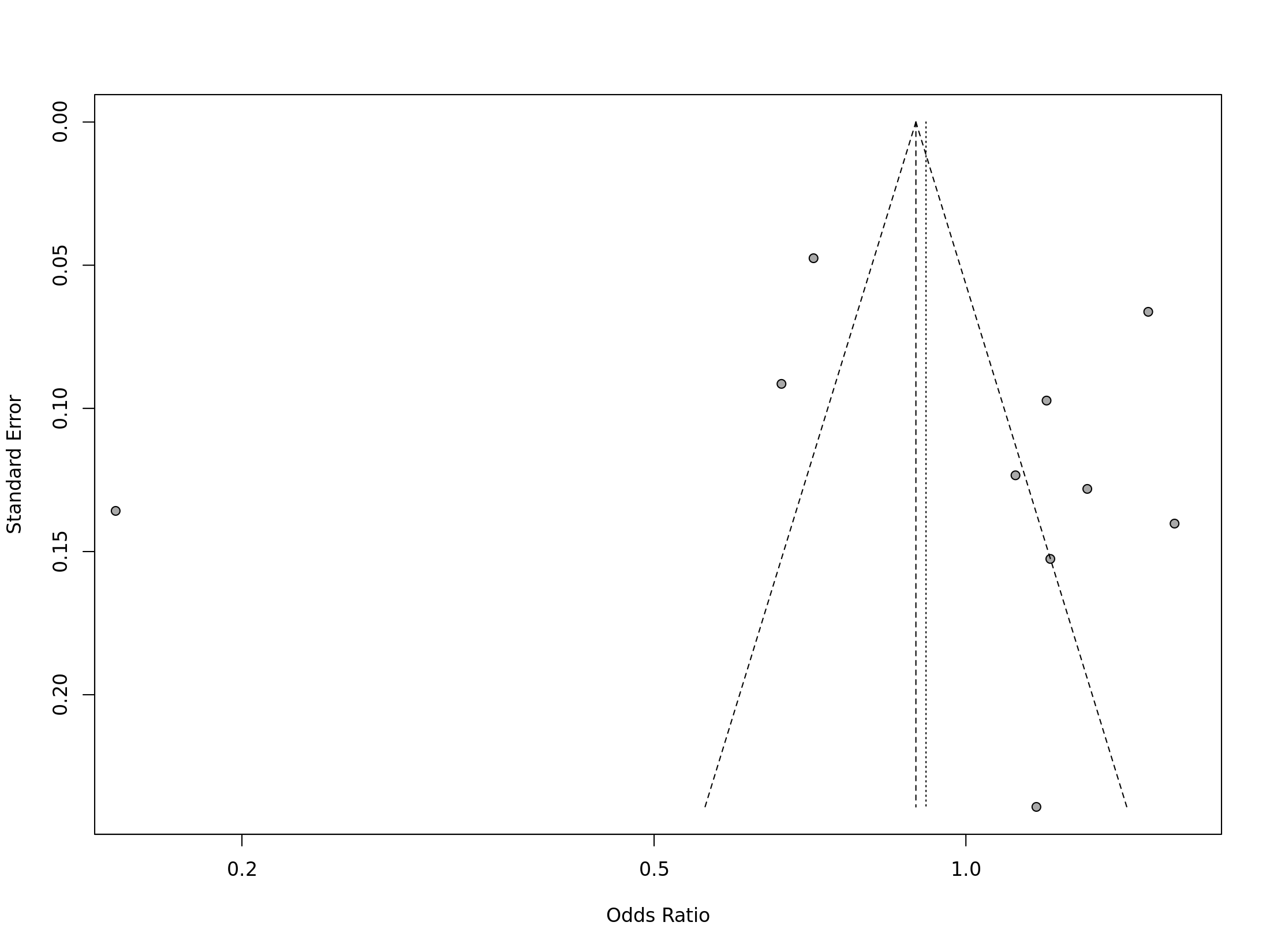
**

**Figure S5. Funnel plot of rs356220 dominant model**

**
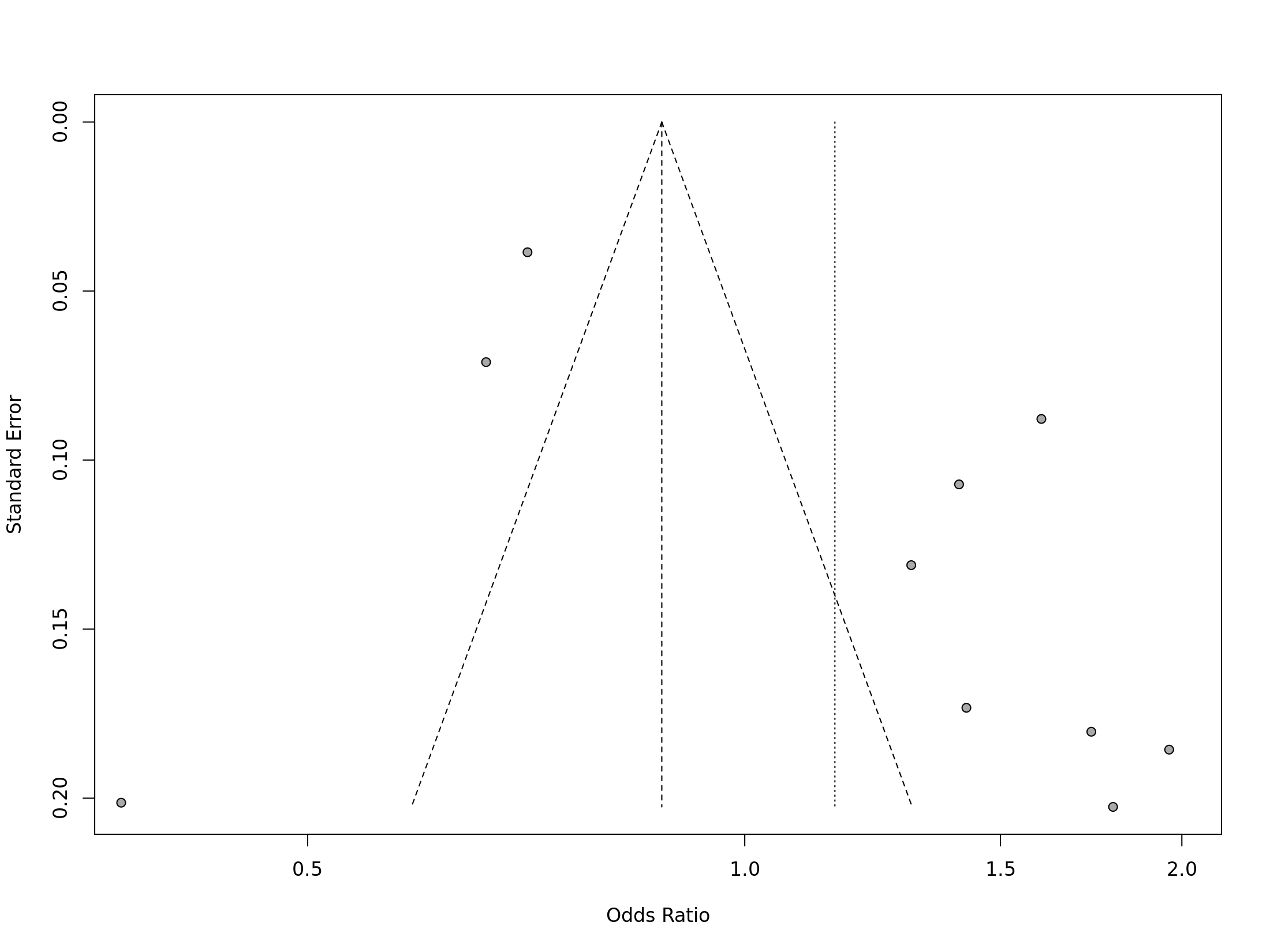
**

**Figure S6. Funnel plot of rs356220 recessive model**
